# EFIN: predicting the functional impact of nonsynonymous single nucleotide polymorphisms in human genome

**DOI:** 10.1186/1471-2164-15-455

**Published:** 2014-06-10

**Authors:** Shuai Zeng, Jing Yang, Brian Hon-Yin Chung, Yu Lung Lau, Wanling Yang

**Affiliations:** Department of Paediatrics and Adolescent Medicine, LKS Faculty of Medicine, The University of Hong Kong, 5 Sassoon Road, Hong Kong, China

**Keywords:** Coding mutation, nsSNP, Amino acid conservation, Functional impact, Evolutionary distance

## Abstract

**Background:**

Predicting the functional impact of amino acid substitutions (AAS) caused by nonsynonymous single nucleotide polymorphisms (nsSNPs) is becoming increasingly important as more and more novel variants are being discovered. Bioinformatics analysis is essential to predict potentially causal or contributing AAS to human diseases for further analysis, as for each genome, thousands of rare or private AAS exist and only a very small number of which are related to an underlying disease. Existing algorithms in this field still have high false prediction rate and novel development is needed to take full advantage of vast amount of genomic data.

**Results:**

Here we report a novel algorithm that features two innovative changes: 1. making better use of sequence conservation information by grouping the homologous protein sequences into six blocks according to evolutionary distances to human and evaluating sequence conservation in each block independently, and 2. including as many such homologous sequences as possible in analyses. Random forests are used to evaluate sequence conservation in each block and to predict potential impact of an AAS on protein function. Testing of this algorithm on a comprehensive dataset showed significant improvement on prediction accuracy upon currently widely-used programs. The algorithm and a web-based application tool implementing it, EFIN (Evaluation of Functional Impact of Nonsynonymous SNPs) were made freely available (http://paed.hku.hk/efin/) to the public.

**Conclusions:**

Grouping homologous sequences into different blocks according to the evolutionary distance of the species to human and evaluating sequence conservation in each group independently significantly improved prediction accuracy. This approach may help us better understand the roles of genetic variants in human disease and health.

**Electronic supplementary material:**

The online version of this article (doi:10.1186/1471-2164-15-455) contains supplementary material, which is available to authorized users.

## Background

Rapid development in sequencing technology and decrease in cost has made it possible to sequence large number of samples on the entire genome or exome. Each individual carries thousands of rare or even private mutations [1, 2]. For cancer tissues, a large number of somatic mutations may also exist in a given tumour. Apparently not all the mutations play functional roles in a disease, and the ones that do may also have different functional impact. It is usually not feasible to characterize large number of mutations experimentally. Analysing ever increasing sequence data *in silico* first and identifying a small number of mutations that are more likely to be involved in diseases for further analysis or experimental characterization is an important task in today’s genetic and genomic studies. Analysing the mutation/polymorphism profiles of protein coding genes in the general population may also help us to better understand the evolutionary history of the genes and their functional roles in human health and diseases.

A number of programs have been developed during the last decade or so and they have played a vital role in predicting the functional impact of human mutations in various genetic, genomic studies [2–12]. Amino acid sequence conservation during evolutionary courses, potential protein structural changes, database annotations, and physicochemical properties of the amino acids involved are among the many features considered in various programs for prediction of functional impact of an amino acid substitution (AAS). Of which, conservation among homologous sequences is considered the most important piece of information in determining prediction accuracy, including programs that take into account of various other features [9].

Despite the enormous progress in this field, accurately predicting damaging AAS from neutral changes is still a challenging task, and most programs have high false positive and false negative rate. Not being able to make use of the full spectrum of homologous sequences and to make use of the information accurately is considered major drawbacks of these algorithms.

In this study, we developed a novel algorithm and a web-based application executing it, EFIN (**E**valuation of **F**unctional **I**mpact of **N**onsynonymous SNPs), to try to make more thorough and more accurate use of conservation information in predicting functional impact of an AAS. Comparison of our program with widely-used programs on various datasets demonstrated significant improvement of EFIN upon others on prediction accuracy.

## Methods

### The work flow of EFIN

**Figure 1:**
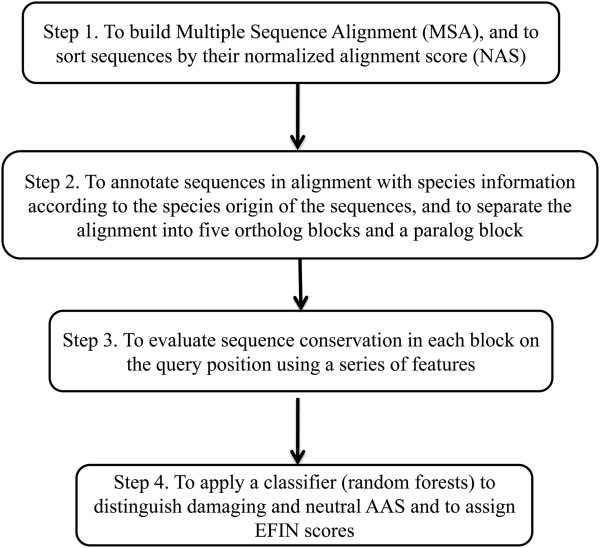
**Flow chart of EFIN.**

The work flow of EFIN is depicted in Figure 1 and is explained in detail below.

### Step 1. Building multiple sequence alignment on the query sequence

EFIN uses BLAST algorithm [13, 14] to identify proteins homologous to the query sequence by searching the UniRef100 database from UniProt (http://www.uniprot.org/). The full length of a query sequence is used for building the multiple sequence alignment (MSA), with a cutoff e-value of 0.0001 and a maximum retrieval of 5,000 sequences for each query process. This is to ensure that the sequences considered are true homologs and an adequate number of such sequences are considered for conservation analysis.

The homologous sequences are then sorted in descending order by their normalized alignment score (NAS), which is the alignment score divided by that of the query sequence itself. The alignment score is an output from BLAST calculated by the amino acid similarity matrix Blosum62. The normalization of alignment score makes it possible to compare proteins of various lengths (see Additional file 1: Method A). The NAS often accurately reflects evolutionary distance between the protein considered and its human ortholog.

### Step 2. Grouping the homologous sequences into blocks

The sequences in MSA are first annotated with taxonomy information of the source species, downloaded from the Taxonomy database [15] in NCBI (ftp://ftp.ncbi.nih.gov/pub/taxonomy/). Based on the taxonomy classification and the evolutionary distance to human, all the sequences in MSA are then grouped into five ortholog blocks and a paralog block. Namely, in descending order, the primate block, the non-primate mammal block, the non-mammal vertebrate block, the invertebrate block, the other species block (including plants, bacteria, and fungi, etc.), and the paralog block.

Briefly, the NAS-sorted MSA is examined starting from the top, usually starting with human sequences and sequences from primates. The first sequence belonging to a different species category (usually from a non-primate mammal, and so on so forth) is marked. According to the evolutionary distance to human, we classified all species into 5 species categories which are primate with human included, non-primate mammal, non-mammal vertebrate, invertebrate and other species such as plant, bacteria, and fungi. The first sequence of each species category in the MSA is the sequence from that category with highest alignment score. Namely, the first primate sequence, the first non-primate mammal sequence, the first non-mammal vertebrate sequence, the first invertebrate sequence, and the first “other species” sequence such as those from plant, bacteria, and fungi.

All the sequences between the two ‘first sequences’ in MSA are then grouped into one block. For example, all sequences between first non-primate mammal sequence (inclusive) and first non-mammal vertebrate sequence (exclusive) are grouped into the non-primate mammal block. The sequences within an ortholog block that should not belong to the same species group are also tagged and moved to the paralog block. For example, protein TP63 and TP73 are paralogs of TP53, and these sequences are usually mixed with orthologs in the MSA for TP53 due to their sequence similarities. TP63 from human (H2QNY5) has comparable sequence similarity to human TP53 to that between human TP53 and fish TP53 (such as H2LPP5). Thus in this process, these TP63 and TP73 sequences will be removed from the non-mammal vertebrate block and will be placed in the paralog block. A mathematical description and an example are also included in the Additional file 1: Methods G and H, respectively.

### Step 3. Evaluation of sequence conservation

Table 1 listed the features used by EFIN, which are explained in detail below.

**Table 1:** **The features used by EFIN**

Name	Description	Value and range
Reference amino acid (AAref)	The reference amino acid of the query position	nominal (A,R,N…V)*
Mutant amino acid (AAmut)	The mutant amino acid of the query position	nominal (A,R,N…V)*
Frequency of reference amino acid (Fref)	Frequency of reference amino acid at the query position in each block	interval [0,1], with 1 means perfect conservation of reference amino acid
Frequency of mutant amino acid (Fmut)	Frequency of mutant amino acid at the query position in each block	interval [0,1], with 1 means that all sequences have the mutant amino acid at the position
Shannon Entropy (H)	Shannon entropy in each block at the query position	interval [0,4.322], 0 means no diversity and larger number means more diversity at the position
NAS of the first sequence in each block (NASfirst)	Normalized alignment score of the first sequence in each block.	interval [0,1], while 1 means identical sequence to the query human protein
Number of sequences in each block (No_all)	Number of total sequences in each block	Interval [0,5000], while 5000 is the cutoff for each MSA
Number of sequences which cover the query position in each block (No_qp)	Number of sequences that cover the query position in each block	Interval [0,5000], while 5000 is the cutoff for each MSA
No_qp/ No_all (RatioNN)	The ratio of No_qp and No_all	Interval [0,1]
Lowest conserved block	The lowest block for which all sequences, together with all the sequences in upper blocks, have the reference amino acid perfectly conserved.	Ordinal (primate block, Non-primate mammal block, non-mammal vertebrate block, invertebrate block, other species block)

*The 20 amino acids in human proteins.

#### Frequencies of reference and mutant amino acid in a block

Frequencies of the reference amino acid (usually wild-type amino acid) or mutant amino acid (changes due to mutations or polymorphisms) at the query position in each block can be calculated as:1

Where *n* is the total number of sequences in that block and A_i_ represents the amino acid of the *i*^*th*^ sequence at query position of the alignment in that block. “*I*” is the indication function.

#### Shannon entropy

Shannon Entropy of the query position of the protein for sequences in a given block is calculated as reported [16, 17]:2

*p*(*a*) stands for the frequency of amino acid *a* at the query position for sequences in a given block, which is calculated in formula (1). AA represents the set of 20 amino acids in human proteins.

#### Evolving rate of a protein

Proteins may evolve at a different rate during evolutionary courses. Some proteins, such as those involved in immune responses, may evolve much faster compared to structural proteins and other housekeeping molecules. Comparison of sequence conservation during evolutionary courses among different proteins may suggest different functional constraints. We used the difference of NAS between the query sequence (human) and the first sequence in each block (except the primate block for which the human sequence is almost always the first sequence) to represent the evolutionary distance between human and other species in different blocks. A larger distance between these sequences compared with those of other proteins might indicate that the protein has been evolving faster, and novel functions may have been developed among the more advanced species. Thus, similar conservation level for proteins that are evolving at a different rate may suggest different functional implications.

#### Number of sequences in each block

This variable describes the total number of sequences in a block. Since some sequences in a block may only partially align with the query protein and may not cover the query position, the number of query-position-covering sequences in each block is a different variable and is also considered here. The ratio of the two (RatioNN) may suggest functional implications of the protein sequences surrounding the query position. Positions outside functional domains tend to have lower RatioNNs than those falling in domains since sequences in functional domains tend to be better conserved.

#### The lowest conserved block

The lowest conserved block is defined as the block for which all its sequences, together with all sequences in upper blocks have the reference amino acid perfectly conserved. Additional file 1: Method H gives an example of the lowest conserved block.

### Step 4. Using random forests as a classifier to evaluate AAS

Random Forests [18] are used as a classifier to distinguish neutral and damaging AAS using the features listed above. Random Forests are an ensemble learning method for classification (and regression), which constructs a multitude of decision trees and outputs the prediction as the majority vote (and average result for regression) from all individual trees. Random Forests is implemented by randomForest package (http://cran.r-project.org/web/packages/randomForest/index.html) in R (http://www.r-project.org/), and the detailed method is also included in Additional file 1: Method D.

### Training and testing datasets

Two datasets were used to train EFIN. UniProt-Swiss-Prot Protein Knowledgebase (referred as Swiss-Prot dataset in this paper) is a dataset containing both known neutral polymorphisms and disease-related mutations, (humsavar.txt, http://www.uniprot.org/docs/humsavar) [19]. It is one of the most comprehensive human protein variant databases and contains 37,331 neutral mutations/polymorphisms and 22,617 disease-related mutations (as of release in January 2013). Potential functional impact of the variants in Swiss-Prot dataset was determined based on literature reports on probable disease associations.

HumDiv [9], a training and testing dataset used in PolyPhen-2, is a dataset with more extreme cases for damaging mutations/polymorphisms compared with Swiss-Prot dataset (http://genetics.bwh.harvard.edu/pph2/dokuwiki/downloads). It contains 5,322 damaging variants known to be causal to human Mendelian diseases, and 7,070 amino acids that only differ between human proteins and their closely related mammalian orthologs, which are considered to be neutral. Damaging variants in the Swiss-Prot dataset include not only casual variants for Mendelian disease but also variants associated with complex diseases, thus covers a much broader range of variants that may have a functional impact. Comparing with Swiss-Prot dataset-trained EFIN, HumDiv dataset trained-EFIN is much more specific on identifying damaging mutations relevant to Mendelian disease. More detailed information about these two datasets can also be found in the Additional file 1: Method C and Additional file 1: Table S2.

EFIN outputs both a prediction (neutral or damaging for the AAS in question) and a score for each query, with the latter being the probability that a mutation is neutral. The smaller the score, the more likely that the query mutation is a damaging mutation. The prediction is based on comparison between the score and the cutoff value. An AAS is considered to be damaging if its EFIN score is smaller than the optimal cutoff value. The optimal cutoff value was determined to obtain the lowest misclassification rate (highest accuracy) for each training set, which is 0.6 for Swiss-Prot-trained EFIN, and 0.28 for HumDiv-trained EFIN in this study.

## Results

### Distance between adjacent sequences within or between blocks

**Figure 2:**
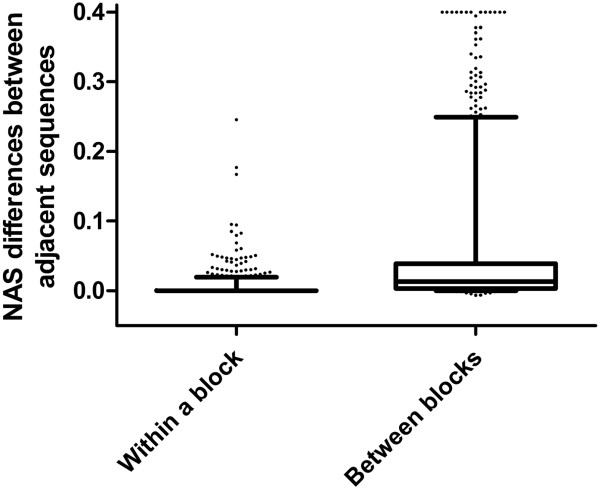
**Box plot of NAS differences between adjacent sequences belonging to either the same block or two adjacent blocks.** NAS differences larger than 0.4 were all treated as 0.4.

Normalized alignment score (NAS) measures similarities between a protein sequence and the querying sequence. To validate the use of the block-wise structure introduced in this study in evaluation of conservation, we compared NAS in two situations between two adjacent sequences in a multiple sequence alignment (MSA): when two adjacent sequences belong to the same block or when they belong to two different blocks (the last sequence of a block and the first sequence in the block next to it). It can be seen that a much greater difference was observed when two adjacent sequences belong to different blocks than when they belong to the same block (Figure 2), using 250 randomly selected pairs for each case. This observation provides a justification for this block-wise approach in sequence conservation analysis.

### Evolving rate of different proteins

**Figure 3:**
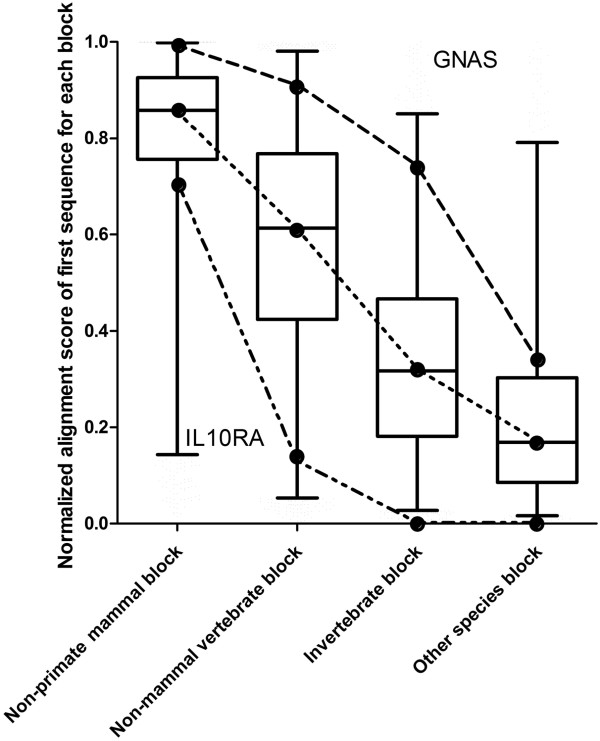
**Distribution of NAS of the first sequence in each block.** Shown are the general distribution of NAS (boxplot) and those from two proteins, IL10RA (dashed line) and GNAS (intermittent dashed line). The general distribution of NAS of the first sequences from each block was generated from randomly selected 12,000 human proteins in UniProt. IL10RA, which encodes a subunit of the interleukin-10 receptor, and GNAS (Guanine nucleotide-binding protein G(s) subunit alpha isoform short), a house-keeping signal transduction molecule, are presented here as examples of different evolving rates of proteins.

NAS of the first sequence in each block was used as one of the features for evaluation of sequence conservation, and it also served as an indicator of evolving rate for a protein during evolutionary courses. As shown in Figure 3, as an example, IL10RA (interleukin 10 receptor, alpha) evolved much faster than most other proteins (shown as the box plot), especially for sequences in non-primate mammal block, suggesting development of novel functions for this gene in mammals. On the other hand, GNAS, a prototype house-keeping signal transduction molecule expressed in all cells, evolved much slower than most other proteins, with near perfect conservation between the human protein and that of other mammals, an indication of extreme functional constraint.

### Relationship between the lowest conserved block and functional impact of an AAS

**Figure 4:**
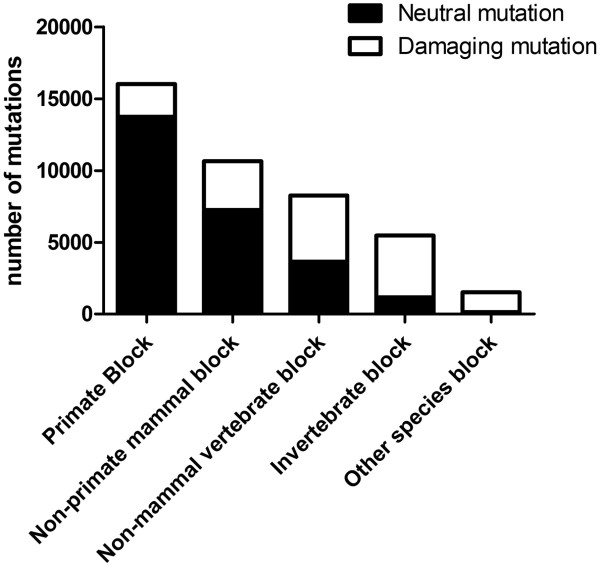
**The ratio of neutral vs. damaging mutations in relationship to the lowest conserved block.** (Paralog block was not considered for this feature).

The lowest conserved block is a novel feature derived from MSA block-wise structure, which calculates for how many consecutive species blocks that the query position is still conserved. The lowest conserved block is one of the most important features for this analysis. Figure 4 shows the ratios of damaging and neutral mutations when different blocks were the lowest conserved block. We investigated this relationship using mutation information from Swiss-Prot dataset. It can be seen that when the primate block was the lowest conserved block, an AAS was more likely to be neutral. When non-mammal vertebrate or lower species blocks were the lowest conserved blocks, an AAS was much more likely to be damaging.

### Comparison of EFIN with other programs

We compared EFIN with the updated versions of five widely-used programs: SIFT (the release of April 2011) [3], PolyPhen-2 (version 2.1.0 released in May 2011) [9], MutationTaster [11], GERP++ (released around May 2011) [10], and phyloP [4]. SIFT program is based on the evolutionary conservation of amino acids within protein families. PolyPhen-2 [9] used eight sequence-based and three structure-based features to predict the impact of a mutation/polymorphism and used naive Bayes as a statistical classifier. MutationTaster considered series of information such as sequence conservation, splicing-site changes, loss of protein features and changes that might affect the amount of mRNA. Furthermore, we also compared EFIN’s performance with GERP++ and phyloP. These two toolsets detect conserved regions and sites by phylogenetic models built according to DNA sequence alignments.

Predictions and scores for tested AAS from SIFT and PolyPhen-2 were obtained using their web-based programs. Scores from GERP++ and phyloP, were retrieved from an annotation database, dbNSFP (v2.0 released in Februrary 2013) [20]. MutationTaster scores and predictions were also extracted from dbNSFP, which were originally queried from the website of MutationTaster. PhyloP scores in dbNSFP were extracted from placental mammal subset of pre-computed scores [21] in the UCSC genome browser website. The value 0 was used as a cutoff to distinguish ‘damaging mutations’ from neutral ones for both phyloP score and GERP++ RS score. Detailed method for retrieving prediction and score from these applications is described in Additional file 1: Method B.

**Figure 5:**
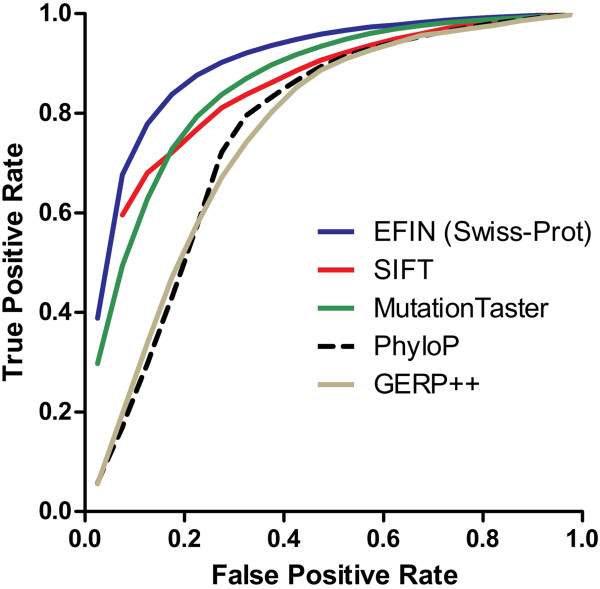
**Receiver operating characteristic (ROC) curves for predictions made by EFIN, SIFT, MutationTaster, phyloP, and GERP++ on the Swiss-Prot dataset.** ROC Curve of Swiss-Prot-trained EFIN, represented as EFIN (Swiss-Prot) in the figure, is the average result of a 10 fold cross-validation.

EFIN, MutationTaster, PhyloP, GERP++ and SIFT were tested on Swiss-Prot dataset. Swiss-Prot-trained EFIN was tested using 10-fold cross-validation with mutations from the same protein being grouped into either the training set or the testing set but not both. Detailed method is included in Additional file 1: Method B. Figure 5 was the receiver operating characteristic (ROC) curve plotted based on data from Additional file 1: Table S1, which listed the true positive rates when false positive rates were fixed for each program. Based on this test, we found that the performance of EFIN compared favourably with that of the other tested tools. Performance was also evaluated by area under the ROC curve (AUC), accuracy (which is the proportion of true results in all the results), specificity, sensitivity and precision (which is the proportion of true positives against all the positive results), and the results were shown in Table 2. In terms of AUC, accuracy, precision and specificity, Swiss-Prot-trained EFIN performed better than the other tools. Although GERP++ and PhyloP are more sensitive than Swiss-Prot-trained EFIN, they both have much higher false positive prediction rates than EFIN.

**Table 2:** Comparison of algorithms tested on Swiss-Prot dataset*

	AUC	Accuracy	Precision	Sensitivity	Specificity
EFIN (Swiss-Prot)**	90.7%(1.0%)	83.7%(0.98%)	86.7%(3.3%)	86.4%(0.9%)	79.5%(3.0%)
GERP++	76.10%	52.80%	45.27%	96.78%	24.36%
PhyloP	76.30%	54.47%	46.18%	96.47%	27.33%
MutationTaster	85.40%	79.47%	69.07%	86.42%	74.98%
SIFT	83.60%	76.58%	67.29%	78.52%	75.32%

*This test is based on a subset of Swiss-Prot, of which mutations can be processed by all these 5 tools, including totally 18660 damaging variants and 28863 neutral variants.

**Swiss-Prot trained EFIN is validated by 10 fold cross-validation, and all these statistical measures are average values. Standard deviation is described within brackets after each measure.

There are a number of mutations that are common among HumDiv, Swiss-Prot and HumVar datasets (Additional file 1: Table S2). Thus, HumDiv-trained EFIN and PolyPhen-2 were tested on Swiss-Prot dataset excluding mutations shared between Swiss-Prot and HumDiv. As shown in Table 3, HumDiv-trained EFIN showed only slightly better performance than HumDiv-trained PolyPhen-2 measured by AUC and accuracy, but had a dramatic advantage in precision. Majority of HumVar mutations overlap with those in Swiss-Prot (Additional file 1: Table S2). Thus, we compared EFIN with HumVar-trained PolyPhen-2 on a subset of Swiss-Prot variants with all HumVar mutations excluded (Table 3). To make a fair comparison on this test, we trained EFIN by the Intersection of Swiss-Prot and HumVar datasets. EFIN showed better performance evaluated by ROC curve, AUC, precision, sensitivity and specificity. ROC curves for these comparisons are depicted in Additional file 1: Figure S1.

**Table 3:** **Comparison of EFIN with PolyPhen-2 on a subset of Swiss-Prot variants**

Tools (Training set)	AUC	Accuracy	Precision	Sensitivity	Specificity
Testing set: Swiss-Prot dataset with HumDiv mutations excluded (36998 neutral variants, 17643 damaging variants)*					
EFIN (HumDiv)	86.96%	80.71%	85.96%	84.96%	72.16%
polyphen2 (HumDiv)	85.26%	78.35%	63.27%	78.59%	78.24%
Testing set: Swiss-Prot dataset with HumVar mutations excluded (15819 neutral variants, 2284 damaging variants)*					
EFIN (Swiss-Prot ∩ HumVar)**	84.91%	71.37%	96.72%	69.60%	83.64%
polyphen2 (HumVar)	80.60%	78.09%	32.28%	67.12%	79.67%

*This test is based on a subset of Swiss-Prot dataset of which mutations can be processed by both EFIN and PolyPhen-2.

**EFIN is trained by intersection of HumVar and Swiss-Prot datasets.

## Discussion

Sequence conservation during evolutionary courses is probably the most important piece of information for evaluating potential functional impact of an AAS. It is also the most widely used information in such efforts. However, how to make full and accurate use of such information is not without difficulties. Most programs measure conservation by counting the sequences that the reference amino acid is conserved, but selecting only representative sequences as counting can be easily skewed by uneven representation of sequences from different species and contamination of databases. This affects the accurate use of conservation information since evolutionary distance is not taken into full account, and limiting the number of sequences used unavoidably causes loss of valuable information.

A common ancestor of human and other primates dates back 23 Myr, and it was 95 Myr for all the mammals and 416 Myr for non-mammal vertebrates, and much farther for invertebrates and other species [22]. For paralogs, the evolutionary distances vary depending on when the new paralogs were originated in evolutionary history. Conservation for a protein among species of different evolutionary distances implies dramatic difference in constraints on protein function and should be taken into full account in functional prediction.

Although programs such as PolyPhen-2 [9] uses information from distant species in evaluating amino acid conservation and MAPP [5] assigns different weights to homologous sequences according to a phylogenetic tree structure, they both have limitations either by not taking full account of evolutionary distance among proteins or by using only limited number of sequences. Making more accurate use of information on sequence conservation among species of different evolutionary distance probably explains most of the improvement by EFIN, which is fulfilled by a block-wise structure. By calculating the conservation features block-wise, the classifier will learn to treat the conservation (or lack of it) from different species differently. The observation that the NAS differences of adjacent sequences in the same block are significantly smaller than those between two adjacent blocks in MSA strongly supports the use of block-wise structure in conservation evaluation (Figure 2). We tested the effect of block-wise features by removing the block structure and treating all the sequences in MSA as a single block. This did result in a dramatic decrease in performance (Accuracy dropped from 0.837 to 0.787; precision decreased from 0.867 to 0.820; sensitivity changed from 0.864 to 0.838; and specificity decreased from 0.795 to 0.707).

Both SIFT and MAPP emphasized the importance of using only remote orthologs but not paralogs in their evaluation, even when this means that there were fewer sequences to be analysed [5]. For PolyPhen (also PolyPhen-2), however, it was argued that using paralogs improves the accuracy of prediction [9]. Here we tried to separate the paralogs from orthologs by grouping the paralogs into a separate block. Thus the pieces of conservation information from both groups can be utilized, yet they were treated differently to reflect potentially different implications on conservation from the two groups.

Overrepresentation of protein sequences from a single species can significantly affect prediction accuracy. For example, Miller et al. [23] described a potential problem for SIFT when a protein was overwhelmingly represented by repeated sequencing and reporting to databases, and the impact of this can be minimized by the block-wise structure adopted in EFIN. Similarly, damages caused by database contamination can also be minimized by this block-wise structure and evaluation process, as the information is extracted from multiple layers independently. For example, human somatic mutations in genes such as TP53 do appear in the ‘nr’ database from NCBI, which may give inaccurate information on sequence conservation (or lack of it). The block-wise structure used by EFIN minimizes the damage caused by repeated sequencing or contamination of databases. As a result, as many homologous sequences as possible can be used during evaluation to maximize information gain.

By searching for one of the most comprehensive protein database (UniRef100 in UniProt), and using a very inclusive threshold to maximize the number of sequences used, EFIN ensures a thorough use of information from homologs, which is quite different from most existing programs. SIFT selects sequences by adding the most similar sequence from a protein database iteratively to a growing collection until conservation in the conserved regions decreases. PolyPhen [24] identifies homologs of the input sequences via BLAST [13] search of the ‘nr’ database, and uses a clustering algorithm and only considers the homologs that belong to a compact cluster that includes the analysed sequence. MutationTaster [11] makes multiple sequence alignment based on amino acid sequences from only ten other species (from chimpanzee to worm). MAPP also only uses a limited number of orthologs in its prediction [5]. The number of sequences used for each query by EFIN is significantly higher than those used by other programs, which is probably an important factor for an improved performance.

Different proteins may evolve at a different rate (as exemplified by the two proteins, GNAS and IL10RA shown in Figure 3). A protein may evolve at a higher rate compared to other proteins, suggesting development of new functions in order to adapt to new environment or to meet functional requirement of the new species. On the other hand, for proteins such as GNAS, there is nearly 80% sequence conservation between the human protein and its orthologs in invertebrate, consistent with a basic house-keeping function for the molecule. Therefore, measuring evolutionary distance among different blocks for each query may be more meaningful than using split time between species during evolutionary courses in general. NAS of the first sequence in each block was used in EFIN to estimate the evolutionary rate and to improve prediction accuracy. Removal of this feature without changing any other features did have a negative effect on prediction accuracy (data not shown).

Theoretically, adding more features may further increase the prediction accuracy of the program. Some features, such as physico-chemical property of amino acid variants and structural information, were used successfully before [5, 24]. Although these features are useful when used alone, they did not significantly improve prediction accuracy after the block-wise conservation evaluation was adopted, suggestive of information overlapping. Detailed evaluation of structural changes is also difficult and may only apply to limited number of proteins with known structures at present. Although detailed consideration of protein features may improve prediction accuracy, as a general prediction tool, making use of only conservation information is a balanced approach between efficiency and accuracy of a program.

SIFT, PolyPhen-2, MAPP and other programs are usually applicable to any proteins for which homologous sequences are available, while EFIN at its present form can only apply to human proteins. Focusing on human proteins, however, has given EFIN the flexibility to make full use of the detailed species information of the homologs. Since EFIN is based on a machine learning method, it is possible or even desirable to use tailor-made training set for different purposes. For example, certain dataset may be more suited for detection of susceptibility variants, while applying to single gene disorders, training set from known causal mutation databases such as HumDiv, OMIM or HGMD [25] may work better. A selected subset of known mutations could also be used for mutation evaluation for a given gene or a group of genes, although this may mean a much smaller training set.

Thus, building on previous endeavours and progresses, EFIN made further improvement on prediction accuracy in this research area of ever increasing importance and may help move us one step closer towards accurate functional prediction of amino acid substitutions in a protein. By no means that EFIN is designed to replace other programs, and it is more of an addition to the set of available tools that have been already widely used in this field. Using various tools to collectively predict the functional impact of a mutation has been proposed by various software [20, 26–28]. The novel approach of block-wise evaluation proposed by EFIN may have added benefits that may help us better understand human variants in our genome.

## Conclusions

To predict damaging AAS, we developed a novel algorithm that tries to make full use of sequencing conservation information by dividing homologous sequences into five ortholog blocks and a paralog block. We used a number of conservation features derived from these blocks and a random forest machine learning method for classification of damaging and neutral amino acid changes. As presented by its receiver operating characteristic curve, the performance of EFIN compared favourably to that of a number of popular prediction tools.

## Electronic supplementary material

Additional file 1: Table S1: Comparison of TPR at different FPR levels for 5 tools tested on Swiss-Prot dataset. **Table S2.** Number of variants in each dataset together with number of variants shared among them. **Figure S1.** Receiver operating characteristic (ROC) curves for predictions made by EFIN and PolyPhen-2. **Methods.** (DOC 999 KB)
