# Supplementary material for: EFIN: predicting the functional impact of nonsynonymous single nucleotide polymorphisms in human genome
Source: BMC Genomics. 2014 Jun 10;15(1):455. doi: 10.1186/1471-2164-15-455 (PMC4061446; doi:10.1186/1471-2164-15-455)
Supplement: Supplementary file 1 — Additional file 1: Table S1: Comparison of TPR at different FPR levels for 5 tools tested on Swiss-Prot dataset. Table S2. Number of variants in each dataset together with number of variants shared among them. Figure S1. Receiver operating characteristic (ROC) curves for predictions made by EFIN and PolyPhen-2. Methods. (DOC 999 KB) [file 12864_2013_6120_MOESM1_ESM.doc]

**Additional file 1**

EFIN: Predicting the functional impact of nonsynonymous single nucleotide polymorphisms in human genome

Shuai Zeng, Jing Yang, Brian Hon-Yin Chung, Yu Lung Lau, Wanling Yang

Department of Paediatrics and Adolescent Medicine, LKS Faculty of Medicine, The University of Hong Kong

**Index**

**Supplementary Table 1.** Comparison of TPR at different FPR levels for 5 tools tested on Swiss-Prot dataset

**Supplementary Table 2.** Number of variants in each dataset together with number of variants shared among them

**Supplementary figure 1.** Receiver operating characteristic (ROC) curves for predictions made by EFIN and PolyPhen-2

**Supplementary Methods**

A. Calculating normalized alignment scores (NAS)

B. Retrieving scores and predictions of other tools and comparison of their performances

C. the Swiss-Prot and HumDiv datasets

D. Training and validation

F. The detailed features used in EFIN

G. Mathematical description of grouping the homologous sequences into blocks

H. Explanation of the general process with an example of ANTXR2 protein

I. Application of EFIN on identifying disease casual mutations

J. Comparison of features used by various tools

**Supplementary Table 1. Comparison of TPR at different FPR levels for 5 tools tested on Swiss-Prot dataset**

|  | **True Positive Rate**  Testing set: UniProt - Swiss-Prot Protein Knowledgebase (Swiss-Prot dataset) | | | | |
| --- | --- | --- | --- | --- | --- |
| FPR | EFIN(Swiss-Prot)* | SIFT | MutationTaster | PhyloP | GERP++ |
| 0.025 | 0.394(0.045) | n/a | 0.2974265 | 0.05740443 | 0.0559087 |
| 0.075 | 0.675(0.041) | 0.5960343 | 0.4939732 | 0.1699641 | 0.1971661 |
| 0.125 | 0.780(0.034) | 0.6802787 | 0.6277153 | 0.2966119 | 0.3382484 |
| 0.175 | 0.838(0.026) | 0.7211683 | 0.7280949 | 0.4299732 | 0.4700548 |
| 0.225 | 0.875(0.019) | 0.7672383 | 0.7930204 | 0.5717248 | 0.5764305 |
| 0.275 | 0.901(0.015) | 0.811129 | 0.8382358 | 0.7224334 | 0.6720142 |
| 0.325 | 0.921(0.014) | 0.837960 | 0.8703431 | 0.7950863 | 0.7424226 |
| 0.375 | 0.936(0.012) | 0.8618435 | 0.897302 | 0.8324009 | 0.802689 |
| 0.425 | 0.949(0.010) | 0.8858199 | 0.9181442 | 0.8660982 | 0.8518858 |
| 0.475 | 0.959(0.009) | 0.9070632 | 0.9347978 | 0.8940088 | 0.8867644 |
| 0.525 | 0.965(0.008) | 0.9224437 | 0.9493845 | 0.9145877 | 0.9095245 |
| 0.575 | 0.972(0.007) | 0.9371919 | 0.9607676 | 0.9300346 | 0.9267268 |
| 0.625 | 0.978(0.005) | 0.9496517 | 0.9699776 | 0.9429094 | 0.9416695 |
| 0.675 | 0.982(0.004) | 0.9598808 | 0.9765443 | 0.9539546 | 0.9563414 |
| 0.725 | 0.986(0.004) | 0.9703979 | 0.9817929 | 0.9642192 | 0.9631534 |
| 0.775 | 0.990(0.003) | 0.9790568 | 0.9858174 | 0.9732491 | 0.970425 |
| 0.825 | 0.993(0.002) | 0.9861047 | 0.9892829 | 0.9806605 | 0.9771539 |
| 0.875 | 0.995(0.002) | 0.9909532 | 0.9921497 | 0.9871516 | 0.9856663 |
| 0.925 | 0.997(0.001) | n/a | 0.9946275 | 0.9936346 | 0.9918701 |
| 0.975 | 0.999(0.000) | 1.000000 | 0.9987138 | 0.9985865 | 0.9973861 |

*: Swiss-Prot dataset trained EFIN. True positive rates of EFIN were calculated as average of 10 fold cross-validation. Standard deviations are described in brackets after true positive rate at each false positive rate level.

**Supplementary table 2. Number of variants in each dataset together with number of variants shared among them**

| **Datasets** | **Neutral variants** | **Damaging variants** | **Total** |
| --- | --- | --- | --- |
| **HumDiv** | 7070 | 5322 | 12392 |
| **HumVar** | 21151 | 22196 | 43347 |
| **Swiss-Prot (updated in January 2013)** | 37331 | 22617 | 59948 |
| **Mutations shared by HumDiv and Swiss-Prot** | 88 | 4719 | 4807 |
| **Mutations shared by HumDiv and HumVar** | 37 | 5307 | 5344 |
| **Mutations shared by HumVar and Swiss-Prot** | 21060 | 20279 | 41339 |


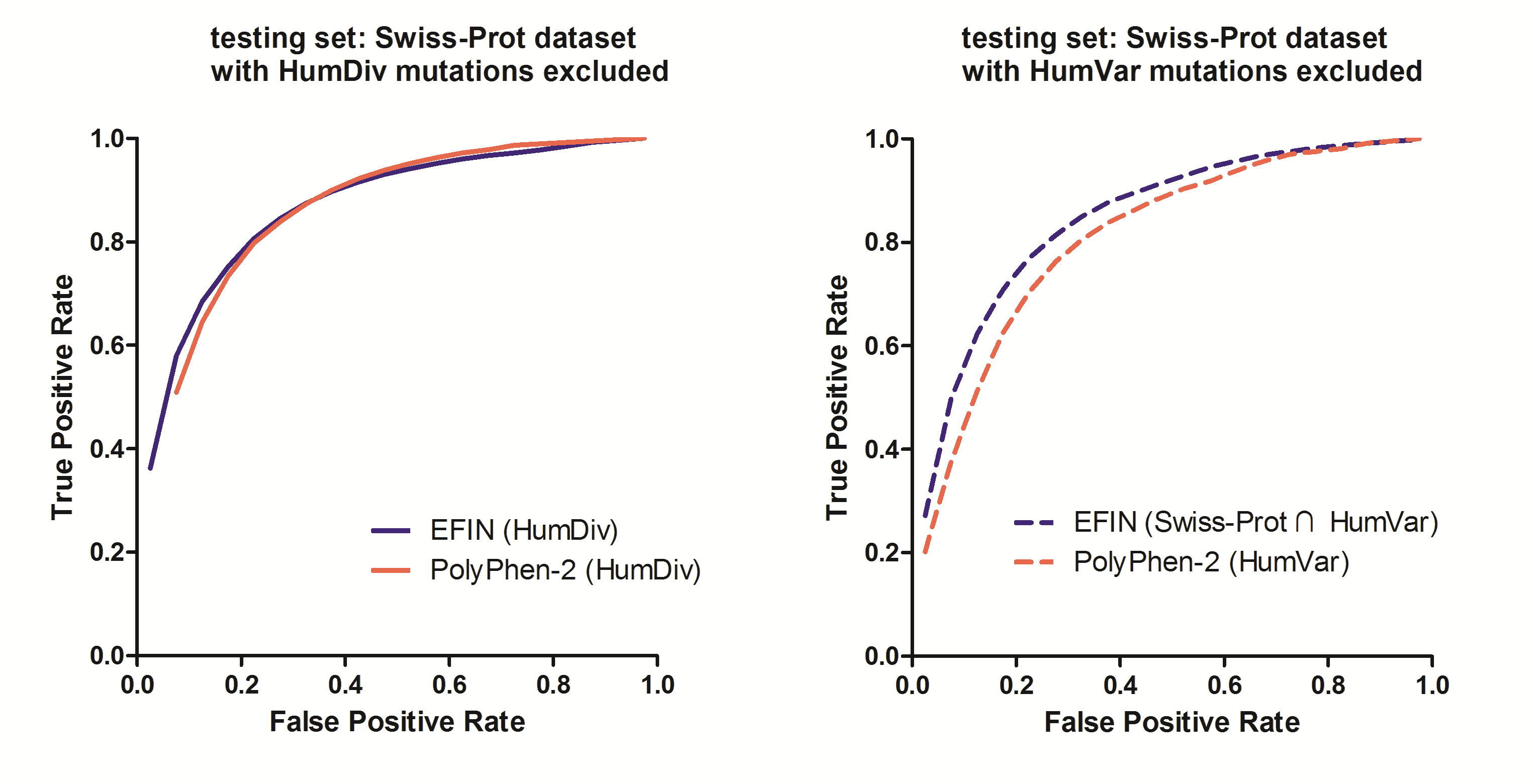


(A) (B)

**Supplementary figure 1. Receiver operating characteristic (ROC) curves for predictions made by EFIN and PolyPhen-2.** (A) ROC curve for EFIN and PolyPhen-2 both trained on HumDiv dataset and tested on a subset of Swiss-Prot dataset with HumDiv mutations excluded. (B) ROC curves for HumVar trained PolyPhen-2 and EFIN which is trained by the intersection of HumVar and Swiss-Prot dataset. Both tools were test on Swiss-Prot dataset with HumVar mutations excluded.

**Supplementary Methods**

***A. Calculating normalized alignment scores (NAS):***

In this work, we note that $Seq'_{n}$ is the n^th^ sequence in MSA, and the 1^st^ sequence ($Seq'_{1}$) is the query sequence itself. For the n^th^ sequence in a Multiple Sequence Alignment (MSA), assuming the aligned length of two proteins is *E*, NAS can be calculated as the following:

$NAS(Seq'_{_{n}})=\frac{\sum_{c=1}^{E} S_{blosum}(A_{nc},A_{1c})-GapCost}{\sum_{c=1}^{E} S_{blosum}(A_{1c},A_{1c})}$ (1)

A_nc_ represents the amino acid of the n^th^ sequence at c^th^ position of the alignment.$S_{blosum}(A_{\text{nc}},A_{1c})$ is the Blosum62 matrix score of amino acid of the n^th^ sequence in MSA at the c^th^ position against the reference amino acid from the query protein at the c^th^ position. This score also takes into consideration of a gap cost (including gap “opening” and gap “extension” cost). The denominator $\sum_{c=1}^{\text{E}} S_{blosum}(A_{1\text{c}},A_{1\text{c}})$is the alignment score of the query protein itself. We sort the MSA by the NAS of the sequences in descending order. The sequences in sorted MSA are: $Seq_{1}$, $Seq_{2}$, $Seq_{3}$…… with $\text{NAS}\left(\text{Seq}_{\text{i}-1}\right)>=\text{NAS}\left(\text{Seq}_{\text{i}}\right)$ for any i>2.

***B. Retrieving scores and prediction of other tools and comparing performance***

**MutationTaster, phyloP and GERP++:** we used dbNSFP to obtain scores and/or predictions from these tools at protein level. dbNSFP is an annotation tool and database that integrates information of DNA, transcript and protein together with scores and predictions from different tools. GERP++ score in dbNSFP is obtained from the precomputed GERP++ Tracks Data. PhyloP score in dbNSFP were extracted from the placental subset of the precomputed phyloP scores provided by the UCSC Genome Browser, which is calculating based on multiple alignments of the 45 vertebrate assemblies to the human genome. We obtained MutationTaster score and prediction from dbNSFP which is originally queried from the web server of MutationTaster. Because MutationTaster need an ENSEMBL transcript ID and snippet (the immediate upstream and downstream from the querying site) for each SNP, the ENSEMBL transcript ID is obtained by Annovar and the snippet is obtained from human reference sequences downloaded from UCSC genome browser.

**PolyPhen-2:** we obtained the PolyPhen-2 score and prediction from website of PolyPhen-2. PolyPhen-2 can use UniProt Accession number as input information, Variants in Swiss-Prot and HumDiv datasets were submitted to PolyPhen-2’s website directly.

**SIFT:** As SIFT recognizes Ensembl ENSP ID rather than Uniprot ID of protein, an ID mapping data from UniProt ( <http://www.uniprot.org/downloads> ) was used to transfer the Uniprot id into ENSP ID. In the ID mapping data, a protein in Uniprot database may have more than one ENSP IDs. We then compare the length of the Uniprot protein with those of its counterparts in Ensembl. If the lengths of those two proteins are not the same, we do not use that mapping relationship. Additionally, some proteins with Uniprot ID do not have their corresponding ENSP ID in Ensembl databases.

***C. Summary of Swiss-Prot and HumDiv datasets***

Impact of variants for Swiss-Prot dataset is assigned according to literature reports on probable disease-association that can be based on theoretical reasons. Swiss-Prot which is probably the most comprehensive non-commercial mutation database is used in our test. There are three kinds of status for mutations in Swiss-Prot database: 'Disease', 'Polymorphism' and 'unclassified'. Only 'Disease' and 'Polymorphism' are used in our test. 'Disease' refers to disease-causing mutations and disease-linked functional polymorphisms. And 'Polymorphism' refers mostly to neutral polymorphism which there is no disease-association report.

HumDiv dataset contains all damaging alleles only with known effects on the molecular function causing human Mendelian diseases in the Swiss-Prot database (if their annotation contains certain keywords implying causal mutation-phenotype) as damaging mutation. Differences between human proteins and their closely related mammalian homologs, are assumed as non-damaging mutations in HumDiv dataset. For detailed method, please see supplementary material in Nature Methods **7**, 248-9 (2010).

***D. Process of training and validation***

When training set and testing set are the dataset, we use 10 fold cross-validations to confirm testing result: Variants in dataset are randomly divided into 10 equal sized subsamples. Mutations belonging to the same gene are forced to be grouped into the same subsample. This helps prevent over fitting. Of the 10 subsamples, one subsample is used as a testing set, and the remaining 9 groups were used as training set. The process is repeated for 10 times with each of the 10 subsamples used exactly once as the validation data testing set.

Random forests can evaluate the ‘importance’ of each feature and implement feature selection in training process. However, by in the ready-made R packages of random forests, the features are evaluated using datasets (Out of Bag dataset) in which mutations from the same gene may be distributed into both training sets and testing sets in the feature selection process(Training process). This may cause potential overfit. Thus, in order to avoid this situation and digging into the ready-made R package, we write an in-house R program to implement the feature selection by a forward step-wise selection process. The forward step-wise feature selection process involves starting with no variables in the model, testing the addition of each variable using a chosen model by the cross-validations described above, adding the variable that improves the model the most, and repeating this process until none improves the model.

***F. The detailed features used in EFIN***

Not all the features in every block are used in the final model. Some features in some blocks may have strong relationship with its counterparts in other blocks, thus those features may not be used in the final model. The table below describes those features used in the final model. For non-block-wise feature, if the feature is used, it is marked ‘Y’; for block-wise feature, the block names are shown if this feature is used in that block.

***Supplementary table F1, detailed features used by EFIN***

| **Name** | **Description** | **Value and range** | **In EFIN model** |
| --- | --- | --- | --- |
| **Reference amino acid (AAref)** | The reference amino acid of the query position | nominal(A,R,N…V)* | Y |
| **Mutant amino acid (AAmut)** | The mutant amino acid of the query position | nominal(A,R,N…V)* | Y |
| **Frequency of reference amino acid (Fref)** | Frequency of reference amino acid at the query position in each block | interval [0,1], with 1 means perfect conservation of reference amino acid | Non-primate mammal block  Non-mammal vertebrate block |
| **Frequency of mutant amino acid (Fmut)** | Frequency of mutant amino acid at the query position in each block | interval [0,1], with 1 means that all sequences have the mutant amino acid at the position | All blocks |
| **Shannon Entropy(H)** | Shannon entropy in each block at the query position | interval [0,4.322], 0 means no diversity; larger number means more diversity at the position | Non-mammal vertebrate block  Invertebrate block |
| **NAS of the first sequence in each block (NASfirst)** | Normalized alignment score of the first sequence in each block. | interval [0,1], while 1 means identical sequence to the query human protein | Non-mammal vertebrate block  Other species block |
| **Number of sequences in each block (No_all)** | Number of total sequences in each block | Interval [0,5000], while 5000 is the cutoff for each MSA | Invertebrate block,  Other species block |
| **Number of sequences which cover the query position in each block (No_qp)** | Number of sequences that cover the query position in each block | Interval [0,5000], while 5000 is the cutoff for each MSA | Non-primate mammal block  Non-mammal vertebrate block |
| **No_qp/ No_all** | The ratio of No_qp and No_all | Interval[0,1] | Invertebrate block |
| **Lowest conserved block** | The lowest block for which all sequences, together with all the sequences in its upper blocks, have the reference amino acid perfectly conserved. | Ordinal (primate block, non-primate mammal block, non-mammal vertebrate block, invertebrate block, other species block) | Y |

***G. Mathematical description of grouping the homologous sequences into blocks***

According to evolutionary distances to human, species are categorized to 6 groups, namely, primate, non-primate mammal, non-mammal vertebrate, invertebrate and other species (like bacteria, fungi and plants). Note *Spe(x)* is the function quantifying taxonomic information of sequence *x*, *Spe(x)* =1, if sequence *x* is from primate. Accordingly, *Spe(x)* = 2, if sequence *x* is from non-primate mammal, 3 for non-mammal vertebrate, 5 for invertebrate and 6 for other species. Note *w_i_* is the set of index on MSA for all the sequences from the *i^th^* species group:

*w_i_ = {j : Spe(j) = i , 1 <= j <=N}, where N is the total number of sequences in MSA*

A ‘first sequence’ of a species groups in a sorted MSA is defined as the first sequence from the species group we meet by reading the MSA from top to bottom. In other words, a ‘first sequence’ is the sequence most similar to querying (human) sequence among sequences from the species group. For i^th^ species group, mathematically, the index of the ‘first’ sequence in a sorted multiple sequences alignment is:

*F_i_ = { j$\in$w_i_ : k$\in$w_i_ , j <= k }*

After that, MSA is divided/grouped by ‘first sequences’ into 6 blocks, 5 ortholog blocks and a paralog block. A sequences between the i^th^ first sequence and i+1^st^ first sequence (F_i_ and F_i+1_) are categorized into either an ortholog block if the sequence and F_i_ are from the same species group. Within each ortholog block, sequences are from the same species group. Mathematically the MSA index of sequences in ortholog block *i* is:

*Blk_i_ = {j : Spe(j) = Spe(Fi), F_i_<= j <= F_i+1_}*

the MSA index of sequences in paralog block is:

*Blk_para_ = {j : Spe(j) < Spe(F_i_), F_i_<= j <= F_i+1_, i = 1,2,3,4,5}*

By doing this, we group the multiple sequence alignment (MSA) into 5 ortholog blocks and the paralog block. The dividing is not only biologically meaningful but also statistically significant which is shown by empirical study (figure 2).

***H. Explanation of the general process with an example of ANTXR2 protein***

Anthrax toxin receptor 2 (Uniref100 id: P58335) is a VWA containing protein translated from ANTXR2 gene. To judge whether amino acid substitutions on certain positions of this protein would be related to disease, we first search Uniref100 database. With the parameters we described, 280 homolog sequences are found. The alignment is then sorted by NAS (normalized alignment score). The sequence in MSA is annotated with species information. Supplementary table H1 shows species information and NAS for top 50 proteins in this alignment.

After that, we scan the MSA from top to bottom, and find out the ‘first sequence’ from each species group in MSA, as shown in Supplementary table H1, marked in italic & red, the first sequences for primate group is P58335 which is from human and the query sequencing itself, and the first sequences for non-primate mammal group is G3SSX2 which is from elephant. The first sequences for non-mammal vertebrate group is K7GIM9 which is from turtle.

Supplementary table H1, species category of top 50 sequences in MSA

| Rank | UniRef100_id | Species | species group* | Alignment Score | NAS |
| --- | --- | --- | --- | --- | --- |
| *1* | *P58335* | *Human* | *1* | *988* | *1.00* |
| 2 | K7B588 | Chimpanzee | 1 | 983 | 0.99 |
| 3 | G7MSZ2 | Rhesus macaque | 1 | 976 | 0.99 |
| 4 | H2PDQ8 | Pongo pygmaeus abelii | 1 | 958 | 0.97 |
| 5 | H2QPS0 | Chimpanzee | 1 | 956 | 0.97 |
| 6 | G1RAV3 | Hylobates leucogenys | 1 | 952 | 0.96 |
| 7 | I2CUV0 | Rhesus macaque | 1 | 947 | 0.96 |
| 8 | A4FUA5 | Human | 1 | 853 | 0.86 |
| *9* | *G3SSX2* | *African elephant* | *2* | *840* | *0.85* |
| 10 | F7EH69 | Rhesus macaque | 1 | 828 | 0.84 |
| 11 | D2GUG5 | Giant panda | 2 | 818 | 0.83 |
| 12 | G3UFW4 | African elephant | 2 | 815 | 0.82 |
| 13 | G1LFC0 | Giant panda | 2 | 805 | 0.81 |
| 14 | J3KPY9 | Human | 1 | 803 | 0.81 |
| 15 | F7AN24 | Horse | 2 | 800 | 0.81 |
| 16 | Q32Q26 | Human | 1 | 799 | 0.81 |
| 17 | K9IKQ7 | Vampire bat | 2 | 799 | 0.81 |
| 18 | I3NHS0 | Ictidomys tridecemlineatus | 2 | 791 | 0.80 |
| 19 | G1SMF8 | Rabbit | 2 | 778 | 0.79 |
| 20 | F1RVC2 | Pig | 2 | 778 | 0.79 |
| 21 | K9J4T3 | Pig | 2 | 778 | 0.79 |
| 22 | G3RQC3 | Lowland gorilla | 1 | 777 | 0.79 |
| 23 | UPI00029D637A | Sheep | 2 | 775 | 0.78 |
| 24 | G5AWK9 | Naked mole rat | 2 | 773 | 0.78 |
| 25 | H0X202 | Garnett's greater bushbaby | 1 | 772 | 0.78 |
| 26 | G1U3Y6 | Rabbit | 2 | 769 | 0.78 |
| 27 | Q08DG9 | Bovine | 2 | 763 | 0.77 |
| 28 | Q00IM8 | Rat | 2 | 763 | 0.77 |
| 29 | E2R0J4 | Dog | 2 | 754 | 0.76 |
| 30 | H0UVU7 | Guinea pig | 2 | 753 | 0.76 |
| 31 | Q6DFX2 | Mouse | 2 | 746 | 0.76 |
| 32 | G3I1L1 | Chinese hamster | 2 | 727 | 0.74 |
| 33 | G1PSH2 | Little brown bat | 2 | 719 | 0.73 |
| 34 | UPI00029DAB48 | Lowland gorilla | 1 | 704 | 0.71 |
| 35 | G3S7I7 | Lowland gorilla | 1 | 696 | 0.70 |
| 36 | G3X1V0 | Tasmanian devil | 2 | 678 | 0.69 |
| 37 | F7DSV6 | White-tufted-ear marmoset | 1 | 671 | 0.68 |
| 38 | F6S811 | Gray short-tailed opossum | 2 | 665 | 0.67 |
| *39* | *K7GIM9* | *Chinese softshell turtle* | *3* | *596* | *0.60* |
| 40 | G1N652 | Common turkey | 3 | 592 | 0.60 |
| 41 | P58335-3 | Human | 1 | 580 | 0.59 |
| 42 | E1C761 | Chicken | 3 | 574 | 0.58 |
| 43 | H9GFZ9 | Green anole | 3 | 562 | 0.57 |
| 44 | K7GIM0 | Chinese softshell turtle | 3 | 556 | 0.56 |
| 45 | B2GUC8 | Western clawed frog | 3 | 545 | 0.55 |
| 46 | F6SXU7 | Western clawed frog | 3 | 543 | 0.55 |
| 47 | H2RQI2 | Japanese pufferfish | 3 | 529 | 0.54 |
| 48 | E7F960 | Chicken | 3 | 529 | 0.54 |
| 49 | A4QP34 | Chicken | 3 | 529 | 0.54 |
| 50 | I3K943 | Nile tilapia | 3 | 528 | 0.53 |

*Species Group, 1 = primate, 2 = non-primate mammal, 3 = non-mammal vertebrate, 4 = invertebrate, 5 = other species

After the ‘first sequences’ for each species group are found, the next step is to group the MSA into different blocks according to evolutionary distance. As is shown in the table table above, all sequences located between P58335 (1^st^ in MSA) and G3SSX2 (9^th^ in MSA) are from primate, and most sequences ranks between G3SSX2 (9^th^ in MSA) and K7GIM9 (39^th^ in MSA) are from non-primate mammals. We group the 1^st^ to 8^th^ sequences into primate block and 9^th^ to 38^th^ sequences into non-primate mammal blocks except sequences marked in green. Those sequences marked in green are probably paralogs, and we move them to paralog block. Sequences in the same block were treated equally.

The following figure represents multiple sequences alignment (MSA) for sequences of primate block, it is easy to find there are some substitutions are neutral by intuition. For example, at position 357 which is marked in red box, amino acid A change to P is probably a neutral mutation. In this case, Frequency of reference amino acid in primate block = 1/8 and Frequency of mutant amino acid in primate block = 7/8, and both Swiss-Prot dataset trained and HumDiv dataset trained EFIN predict this mutation as damaging with quite high confidence (EFIN score: 1 and 0.978 individually).

However this does not indicate that at that position amino acid substitution from A to other amino acids are neutral, other information is still necessarily needed to make a judgment. By applying a variety of features on different blocks, EFIN quantitatively evaluates and weights different aspects of information from the full span of evolution spectrum.

The 8^th^ sequence A4FUA5 in MSA, is an isoform of Anthrax toxin receptor 2 protein (P58335), due to mechanism of alternative splicing. Because both the isoform and querying protein are translated from the same gene, this isoform possibly shares same domains with the querying protein. The conservation on those shared domains is valuable. So we do not exclude this sequence from primate block.


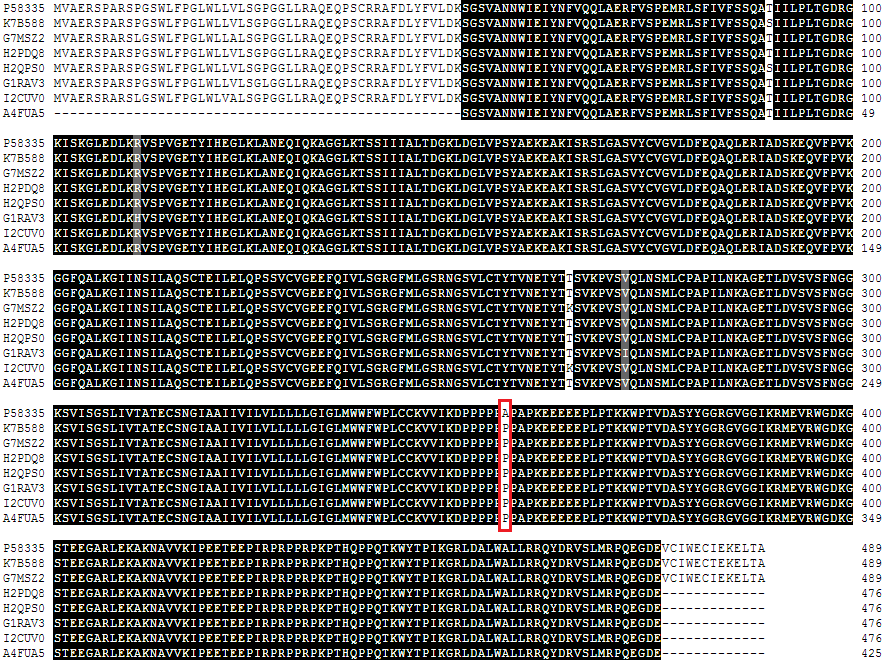


Supplementary figure H1, Alignment in primate block. Some obvious neutral substitution can be evaluated from this graph. For example, at position 357(red box), substitution of amino acid from A to P is more likely to be a neutral mutation.

The following figure (Supplementary figure H2) shows some sites are more conserved than other sites. if substitution of reference amino acid G by D at position of 105 (marked in red box in this figure) is a well documented and reported to be related to Juvenile hyaline fibromatosis (JHF) [MIM: 228600]. The lowest conserved block for this position is invertebrate block (not shown in this figure). Both Swiss-Prot dataset trained EFIN and HumDiv dataset trained EFIN predict it as damaging mutations with highly confidence (EFIN score: 0.064 and 0.008 individually). Considering lowest conserved block, position 87(marked with blue arrow) is conserved to non-primate mammal block. Position 106, which is marked with black arrow, is only conserved in primate block. Position 100 marked by red arrow is not conserved in any ortholog block. For position 87 which is marked with blue arrow, the lowest conserved block is non-primate mammal block. Judging from this aspect, position 87 is more conserved than position 106. And position 106 is more conserved than position 100.


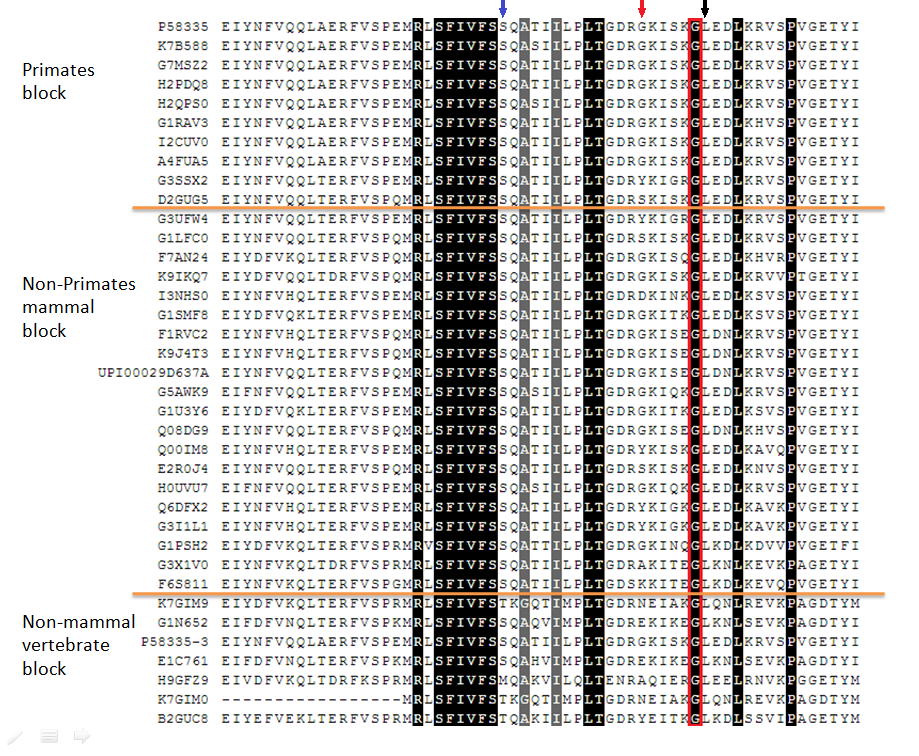


Supplementary figure H2, sequences alignment segment for primate block, non-primate mammal block and part of non-mammal vertebrate block. The orange lines are the boundaries of blocks. Four example query positions, 105, 100, 87 and 106, are marked by red box, red arrow, blue arrow and black arrow individually.

***I. Application of EFIN on identifying disease casual mutations***

Inflammatory bowel disease is well recognized for its genetic involvement in pathogenesis. In this example, we replicated the process of identifying candidate disease casual nsSNPs of our previous research on Crohn’s disease [1] with EFIN. Whole exome sequencing was performed to detect the mutations in the samples from one family with a child suffering from Crohn’s disease.

After exome sequencing data were mapped to genome reference using MAQ, a total 10463 homozygous and 14590 heterozygous SNPs are identified by Samtools. 90 homozygous SNPs and 1125 heterozygous mutations are kept after filtering common SNPs. We further applied EFIN on those SNPs, only keep those damaging mutations predicted by EFIN and located in candidate genes which are genes previously reported related to crohn’s disease. Considering genotypes, two compound damaging heterozygous mutations (supplementary table I1) are found in IL10RA genes. After the potential disease casual mutations are found, web experiments are performed to confirm our discovery and study the biological mechanism behind the observations.

According to figure 3, measured by NASfirst, IL10RA is a fast evolved protein, which may suggest this protein could play a specific role in advanced animal like primate. Regarding to lowest conserved block, both locations were conserved to non-mammal vertebrate block. Substitution in conserved positions in a fast evolved protein, probably will cause lost of protein function because of interfering with structural stability of the protein. These two sites are less likely human specific functional sites.

By doing wet-experiments, we found that these mutations seriously impaired IL-10-induced suppression of inflammatory responses and STAT3 activation. The mechanism involved the mutations abrogating IL-10R1 activation upon IL-10 stimulation, despite normal expression level of IL-10 receptors and IL-10 binding. Reconstitution of wild-type IL-10RA in patient’s cells restored IL-10R function, including IL-10-induced activation of STAT3 and expression of SOCS-3.

Supplementary Table I1, disease casual mutations found by EFIN

| **Location** | **Ref** | **mut** | **EFIN(Swiss-Prot) Score** | **EFIN(Swiss-Prot) Prediction** | **EFIN(HumDiv) Score** | **EFIN(HumDiv) Prediction** | ***Lowest conserved block** |
| --- | --- | --- | --- | --- | --- | --- | --- |
| **84** | T | I | 0.18 | Damaging | 0.006 | Damaging | non-mammal vertebrate block; |
| **101** | R | W | 0.356 | Damaging | 0.002 | Damaging | non-mammal vertebrate block; |

***J. Comparison of features used by various stools***

| **Tool:** | **Species information** | **orthologous/ paralogous sequences** | **Structural information** | **Physicochemical property** |
| --- | --- | --- | --- | --- |
| EFIN | MSA is grouped into different species blocks. Information from different species blocks is treated separately | Both orthologous and paralogous sequences are used.EFIN automatically classifies orthologs and paralogs and treats them differently in later steps | - | - |
| SIFT | - | Both orthologous and paralogous sequences. SIFT automated determines which sequences to use for building MSA, and a majority of orthologs and a few paralogs may be used. Those selected orthologs and paralogs are treated equally. | - | - |
| PolyPhen-2 | - | Both orthologous and paralogous sequences. PolyPhen-2 automated determines which sequences to use for building MSA, and both orthologs and paralogs may be used. Those selected orthologs and paralogs are treated equally. | Considered relevant features of every query position | Considered relevant features of every query position |
| MutationTaster | Only sequences from 10 selected animal species are used to build MSA | Orthologous sequences from 10 animal species are used for conservation analysis | Considered if the querying position is annotated with relevant features in Swiss-Prot database | Considered if the querying position is annotated with relevant features in Swiss-Prot database |
| GERP++ | Only sequences from selected vertebrate species are used to build MSA | Orthologous sequences are used in the web-based version (embedded in UCSC genome browser) | - | - |
| PoyloP | Only sequences from selected vertebrate species are used to build MSA | Orthologous sequences are used in the web-based version (embedded in UCSC genome browser) | - | - |

***Reference:***

1. ***Mao H, Yang W, Lee PP, Ho MH, Yang J, Zeng S, Chong CY, Lee TL, Tu W, Lau YL. Genes Immun. 2012 Jul;13(5):437-42. doi: 10.1038/gene.2012.8. Epub 2012 Apr 5. Exome sequencing identifies novel compound heterozygous mutations of IL-10 receptor 1 in neonatal-onset Crohn's disease.***
